# A novel PRKDC mutation caused B lymphocytes V(D)J rearrangement disorder in the SLE-DAH like symptoms patient

**DOI:** 10.1186/s12969-023-00840-9

**Published:** 2023-08-14

**Authors:** Hongwei Li, Yawen Zhang, Biyun Zhang, Dehui Chen

**Affiliations:** 1 Department of Pediatrics, The First Affiliated Hospital of Guangzhou Medical University, 151 Yanjiang Rd, Guangzhou, 510120 China; 2 State Key Laboratory of Respiratory Disease, National Clinical Research Center for Respiratory Disease, Guangzhou Institute of Respiratory Health, The First Affiliated Hospital of Guangzhou Medical University, Guangzhou, China

**Keywords:** PRKDC, Mutation, Systemic lupus erythematosus, Immunodeficiency

## Abstract

**Background:**

Analyzed the clinical features and treatment process of the patient suffering from immunodeficiency with systemic lupus erythematosus(SLE)-like syndrome in a novel mutation of PRKDC.

**Case presentation:**

The patient had multiple positive auto-antibodies, chest CT and bronchoscopy showed Diffuse alveolar hemorrhage(DAH), and psychiatric symptoms showed brain atrophy by magnetic resonance imaging (MRI). Whole exon sequencing showed that novel complex heterozygous mutations of PRKDC gene (C. 1777 − 710_1777-709INSA (IVS16/IC16), C.1337T > A(p.Phe446Tyr). The mature B cell (CD19 + CD27 + CD38 dimIgD IgM-) were absent. The treatment of high-dose methylprednisolone (MP) and cyclophosphamide(CTX) can quickly relieve the symptoms of the patient.

**Conclusion:**

We described the case of an infant immunodeficiency with SLE like-syndrome, which may cause by PRKDC mutation, treated successfully with high-dose MP and CTX.

## Introduction

Childhood systemic lupus erythematosus (cSLE) has been a common autoimmune disease in children, mainly characterized by multi-system chronic lesions, including skin, joint, kidney, nerve, cardiovascular, and lung damage, accompanied by extensive auto-antibody formation [1, 2]. Diffuse alveolar hemorrhage (DAH) syndrome is a very serious and fatal interstitial lung disease with an average mortality of 50%, caused by autoimmune diseases, especially SLE [3–5].

SLE has been mainly related to the immune dysfunction of B cells. Abnormal activation of B cells can generate multiple auto-antibodies, leading to the formation of immune complexes [6]. PRKDC encodes DNA-dependent protein kinase (DNA-PK),which is abundantly expressed in almost mammalian cells [7, 8]. Studies show that PRKDC mutation influenced production T/B cells and V(D)J rearrangement, which act as major triggers of auto-antibodies and autoimmune disease production [9, 10]. PRKDC mutation is seldom reported on autoimmune diseases and no related studies on SLE, especially for the treatment and prognosis of SLE with this gene mutation.

Here, we found a novel compound heterozygous mutation of PRKDC in a male cSLE for the performances of severe acute diffuse alveolar hemorrhage, multiple positive auto-antibodies, psychiatric abnormalities and multiple pathogen infections. Both parents were found to be heterozygous for the mutation. Aggressive plasma exchange, high-dose methylprednisolone pulse combined with cyclophosphamide (CTX) immunosuppression therapy significantly improved the clinical symptoms of the children. These results suggested that PRKDC mutations may induce T/B cell dysfunction and immune leakage, promoted the formation of auto-antibodies, which caused severe SLE with DAH symptoms.

## Case presentation

An 8-month-old male child was admitted to the hospital because of cough and hemoptyses for more than one month with butterfly erythema and hepatomegaly. An episode of acute onset of alveolar hemorrhage (hemoglobin 8 g/L, Fib 0.5 g/L), pulmonary infection (Enterobacter cholera, Pseudomonas Aeruginosa, Enterobacter cloacae, Mycoplasma pneumoniae, Parainfluenza virus. Acinetobacter and oligoxomonas maltophilia were detected in alveolar lavage fluid with Next Generation Sequencing(*NGS)*, hypoxemia and psychosis were presented. Personal history: The patient was a full-term natural birth baby, and had retarded psychomotor development manifested as dysphasia and poor active grip with regular rehabilitation treatment at the age of 4 months old. Family history: His father worked in the administrative department of electronic components waste recycling. The parents and the sister were in good health. His father’s antinuclear antibody spectrum showed suspicious positive anti-SM antibody and weak positive anti-RNP/Smith antibody. The mother and the sister were negative in routine of auto-antibodies, immunity and hematuria detecting. Past history: He had been hospitalized twice for bronchopneumonia and moderate anemia. Laboratory text of autobiographies showed, positive antinuclear antibody (ANA) (nuclear granular + cytosolic) 1:640, anti-double, stranded DNA (dsDNA) and others were detect in the patient.

**Fig. 1:**
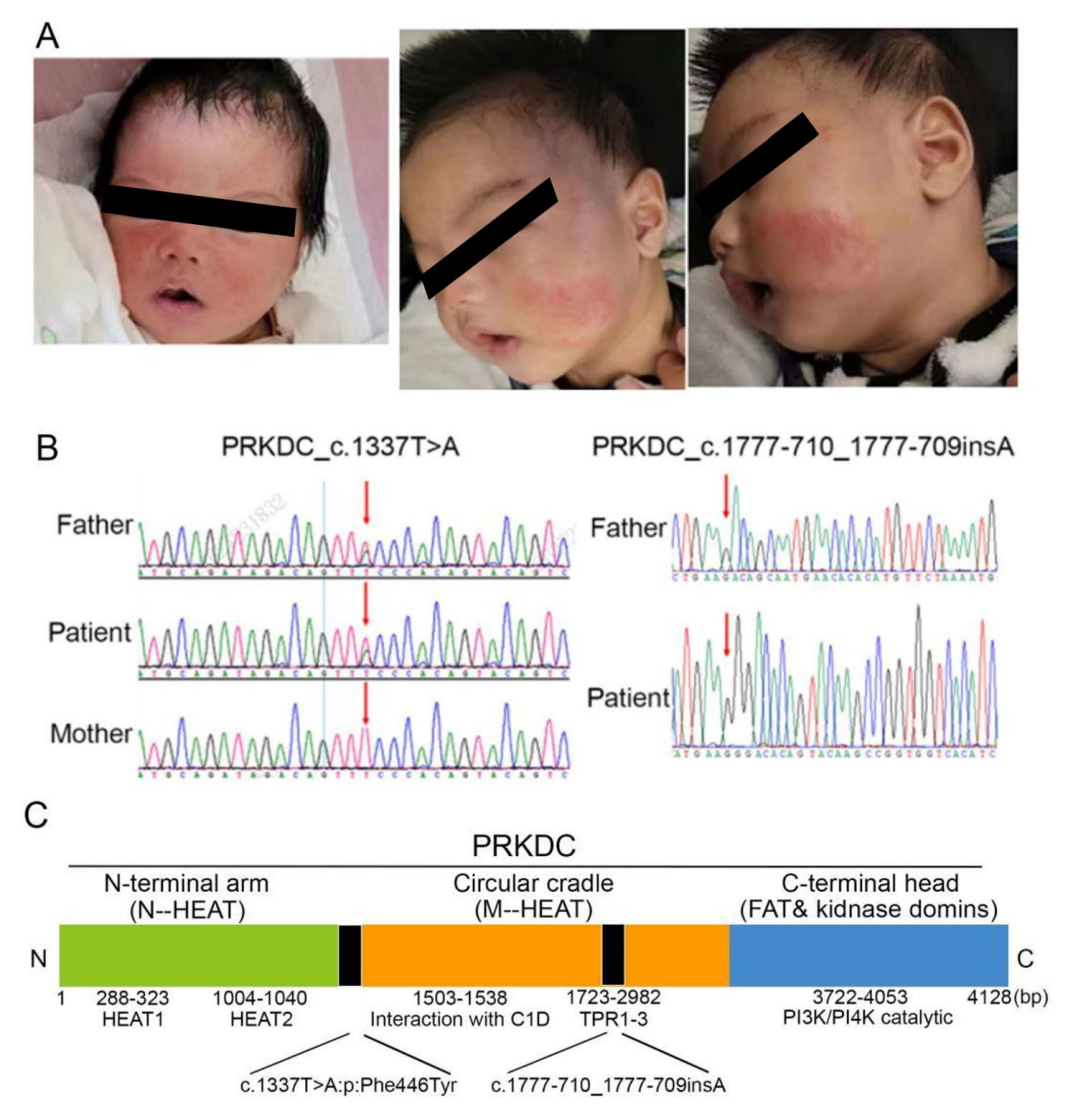
Distinctive annular erythematosus skin lesions and sanger sequencing of compound heterozygous mutation in the patient. **A**. Distinctive annular erythematosus skin lesions of the patient. **B**. Sanger sequencing showed the compound heterozygous mutation of the patient. **C**. PRKDC gene characteristics and domain distribution

**Fig. 2:**
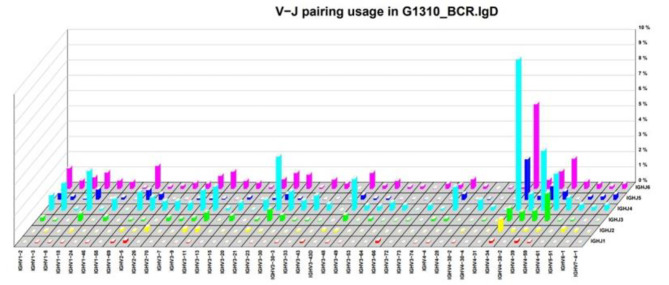
B-cell receptor oligoclonal repertoire in PBMC of the patient with PRKDC mutation. Quantitative analysis of immunoglobulin polymorphism (heavy chain of BCR) of the patient, overrepresentation in IgD mono-clone and the quantification data.

The Whole-exome showed novel compound heterozygous mutation of PRKDC was detect in the patient (Fig. 1), which had been reported to be pathogenic for this variant to involve in the pathogenesis of immunodeficiency type 26 [9]. The CD19 + CD27 + CD38dim IgD/IgM of classical immunoglobulin class conversion B lymphocytes decreased significantly which be converted from IgM to IgG, IgA, IgE, etc. [11] (Table 1). The polymorphism of B-cell receptor (BCR) and T-cell receptor(TCR) were limited in patients with PRKDC mutations (Fig. 2). Above the result present the patient had the deficiency in the VDJ rearrangement with secondary immune disorder.

**Table 1:** Detailed immune function analysis of the patient with PRKDC mutation

Cell type	Cell phenotype	Value	Reference value
B lymphocytes	CD19 + CD3-	16.13	3.80–21.50
Transitional B lymphocytes	CD19 + CD27-CD38high IgM + CD24+	0.09	0.10–6.30
Primary B lymphocytes	CD19 + CD27-IgD+	92.03	17.90–75.10
Marginal zone B lymphocytes	CD19 + CD27 + IgD+	3.04	3.80–35.60
Memory B cells	CD19 + CD27 + CD38dim	3.23	11.00-46.60
Classic immunoglobulin class conversion B cell	CD19 + CD27 + CD38dimIgD-IgM-	0.00	4.60–35.50
Non-immunoglobulin class conversion B cell	CD19 + CD27 + CD38dimIgM+	2.28	1.90–23.70
Plasmoblast	CD19 + CD27highCD38highIgD-IgM-	0.19	0.30–7.80
CD21low B lymphocytes	CD19 + CD38lowCD21low B cells	2.47	1.90–19.30

**Fig. 3:**
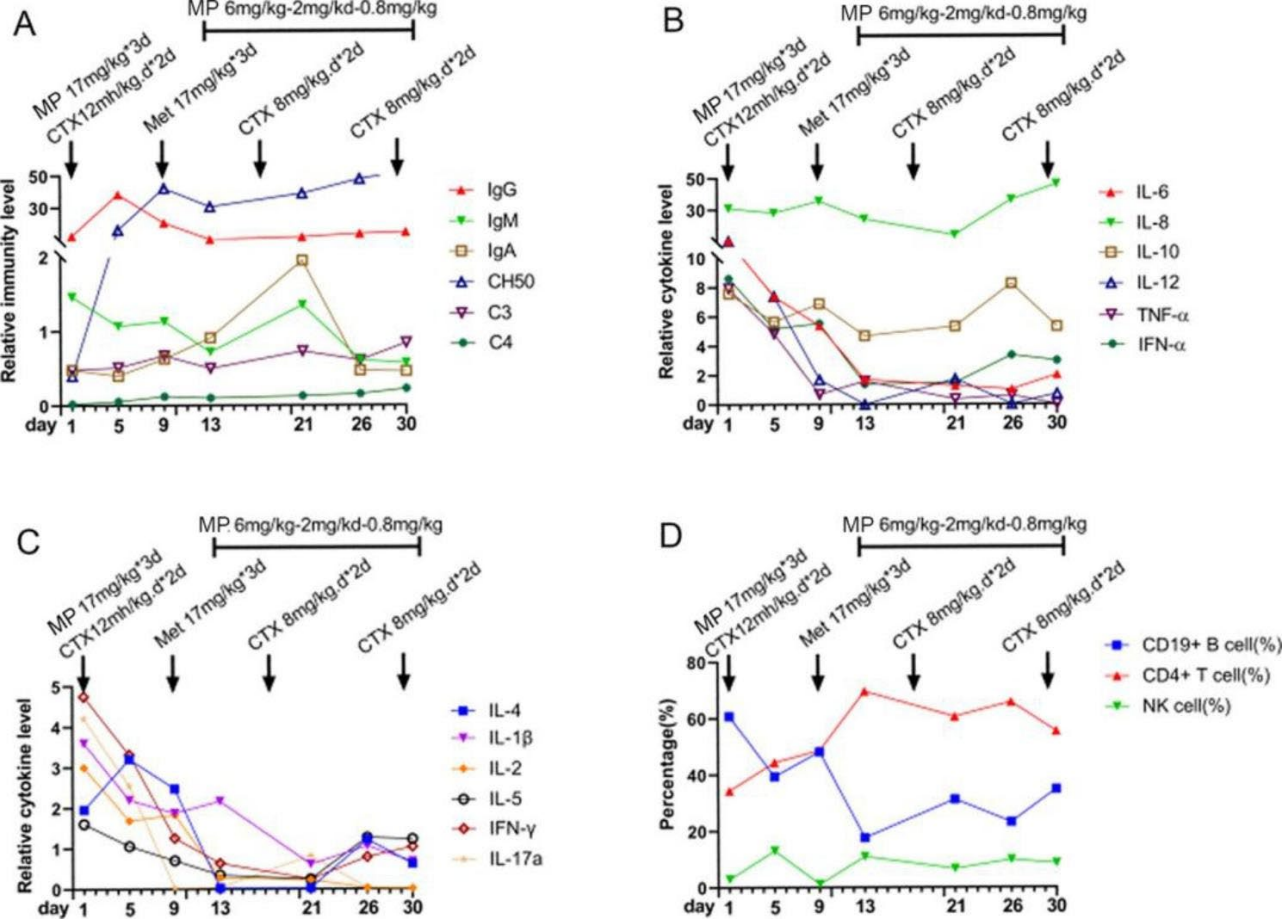
Changes of immunoglobulin, cytokines and lymphocyte ratio before and after treatment

**Fig. 4:**
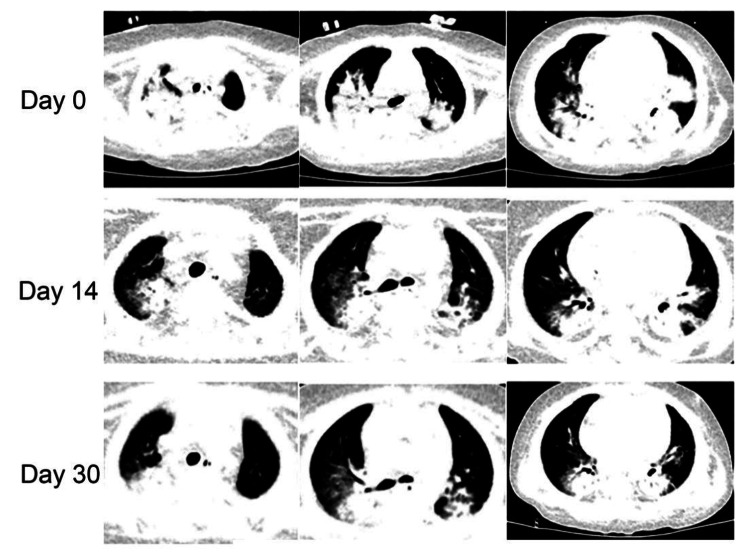
High-resolution chest CT shows pulmonary inflammation and bleeding before and after treatment of the patient

**Fig. 5:**
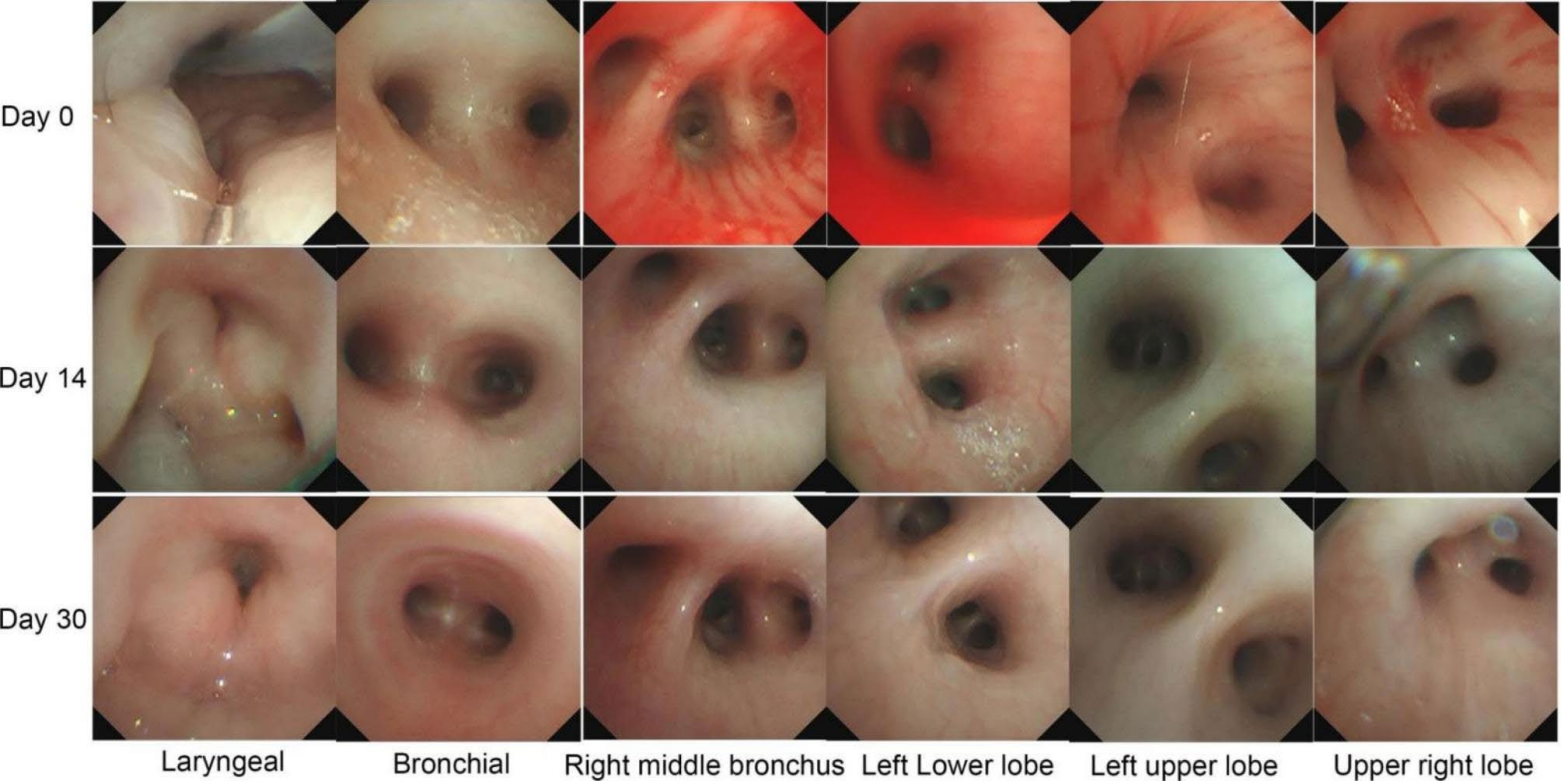
Bronchoscopy showed intratracheal bleeding and inflammation before and after treatment of the patient

**Fig. 6:**
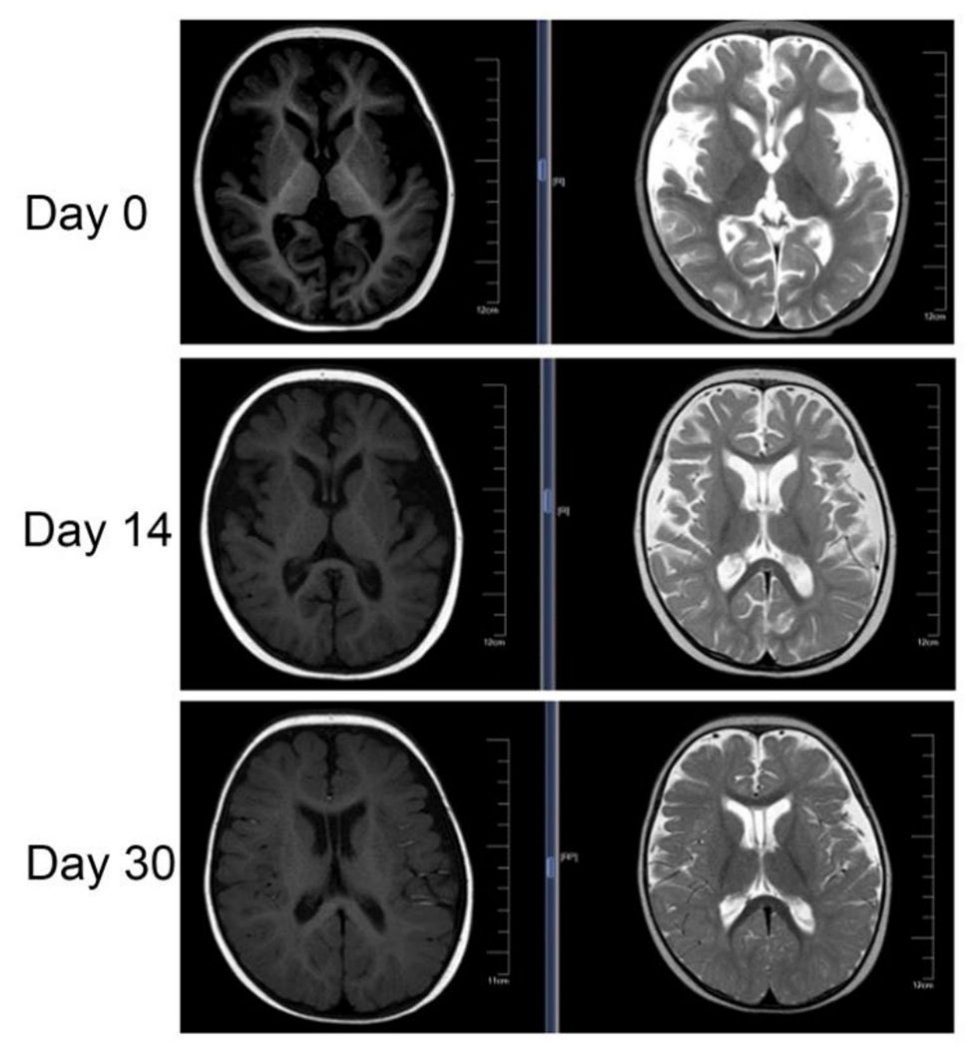
Magnetic resonance imaging showed brain atrophy before and after treatment of the patient

The treatment was given MP combined with CTX immunosuppression, and the dose was adjusted according to the ratio of T/B/NK cell subsets (Fig. 3), supplemented with plasma exchange three times to remove auto-antibodies and inflammatory mediators, and anti-infection treatment were performed with cefoperazone sodium and sulbactam sodium, teicoplanin, azithromycin and voriconazole according to the etiological examination. Finally, the patient’s blood oxygen and hemoglobin were stable, and pulmonary exudation was improved obviously in assessment of high-resolution chest CT (Fig. 4). Chest High-resolution CT showed the imaging changes of DAH (Fig. 5). Bronchoscope record: hemorrhagic diffuse alveolar in left and right side of the bronchial (Fig. 6).

## Discussion and conclusions

SLE was proved to be a typical autoimmune disease and the damage to B cell tolerance checkpoints may be the main reason for the production of auto-antibodies. Antibodies, as secretory Immunoglobulin (Ig), are produced by B lymphocytes and are Y-type protein complexes composed of IgH and IgL pairs connected by disulfide bonds. IgH and IgL have variable and constant regions, respectively, in which the variable region specifically recognizes and binds antigens. Variable region coding genes are produced by V (D) J recombination. In this case, we found the DAH as the first episode symptom of cSLE with genetic compound heterozygous mutation in PRKDC gene. PKCDC regulated apoptosis and proliferation of B cells and it had been reported that mutation of PRKDC can cause type 26 immune deficiency (Table 2), mainly manifested by impaired differentiation of T and B cells, due to DNA-PKCs deficiency and V(D)J rearrangement obstruction [12].

**Table 2:**
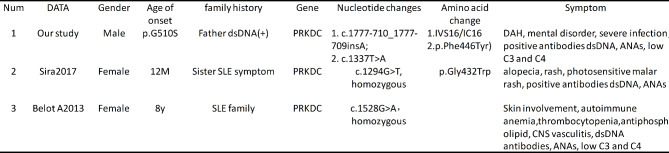
Clinical characteristics and genetic analysis in our study and the reported cases with PRDDC mutation

At the early stage of the disease, we found that the patient presented with nervous system lesions such as dysphoria, irritability, hypertonia and other neurological manifestations which was indicated as brain atrophy by MRI. After treatment with MP and cyclophosphamide, the above symptoms disappeared and brain atrophy resumed. In this patient, Brain atrophy may be associated with the development of symptoms of lupus of the nervous system. A Kalinowska-Lyszczarz reported that the same classifiers were identified in a subgroup analysis that included patients with a short disease duration. In SLE brain atrophy was the main determinant of brain volume. Different correlation patterns between volumetric and clinical data may suggest that is mostly associated with age in SLE.

DNA PKcs-the key partners of autoimmune regulator play an important role in regulating human autoimmune responses, especially in systemic lupus erythematosus. Anne-laure Mathieu found that PRKDC mutation reduced the level of DNA-PKCs, causing autoimmune diseases [9]. In addition, the accumulation of anti-Ku and DNA-PKCs antibodies related to the non-homologous DNA end-joining pathway were detected in SLE patients with PRKDC polymorphism [13–15]. Above all, we suggested the PRKDC mutation can causes the occurrence of SLE.

## Conclusions

Here we described the case of an infant immunodeficiency with SLE like- syndrome, which cause by PRKDC mutation, treated successfully with high-dose MP and CTX. The novel compound heterozygous mutation of PRKDC may be relate to dysregulates B-cell proliferation, promotes auto-antibody formation.

## Data Availability

The datasets used and/or analyzed during the current study are available from the corresponding author on reasonable request.
